# Multiple Chemical Sensitivity: A Clinical Perspective

**DOI:** 10.3390/brainsci14121261

**Published:** 2024-12-16

**Authors:** Louis Jacques

**Affiliations:** 1 Clinic of Occupational and Environmental Medicine, Montreal University Hospital Center, Montreal, QC H2X 0C1, Canada; louis.jacques.med@ssss.gouv.qc.ca or louis.jacques@umontreal.ca; 2 Department of Medicine, Faculty of Medicine, Montreal University, Montreal, QC H3T 1J4, Canada

**Keywords:** multiple chemical sensitivity, comorbidities, emotional traumas, anxiety, stress response, autonomous nervous system, psychotherapy

## Abstract

Objective: The etiology of multiple chemical sensitivity (MCS) is still debated, which is an obstacle to assessing treatment options. An analysis of the scientific literature combined with the clinical experience can suggest some avenues. Methods: The etiology of MCS and its underlying mechanisms were reviewed from the scientific literature to identify the main factors contributing to its development. The results of the studies involving biomarkers and cerebral imaging techniques on MCS subjects were compared with those performed on subjects having the comorbidities of MCS. From the scientific literature and the experience in a clinical setting in occupational and environmental medicine, distinct types of MCS were looked for, with the application of the underlying mechanisms. The potential effectiveness of available treatments was also reviewed. Results: Among many factors, unresolved emotional traumas causing chronic and acute stress reactions play an important role in the development of MCS and can be the basis for effective treatment. We identified three types of clinical presentations, called the accidental type, following a toxic exposure causing an associated emotional trauma, the associative type, following a repeated innocuous exposure in a threatening context, and the developmental type, following a traumatic childhood/adolescence causing hypervigilance and chronic stress/trauma-related disorders. We presented real cases to illustrate these types and the mechanisms behind their development, as well as effective resolution. Conclusions: MCS and its comorbidities could be treated effectively when the underlying emotional trauma(s) are targeted using trauma-focused psychotherapy and other therapies. Diagnostic criteria, principles of treatment and prevention, and avenues for research were derived from this analysis.

## 1. Introduction/Objective

Multiple chemical sensitivity (MCS) is the most frequent subtype of idiopathic environmental intolerance syndrome (IEI), also called environmental hypersensitivity or environmental illness, among other names. In MCS, the intolerance to chemical odors, and sometimes to other types of odor, is characterized by an immediate reaction to apparently innocuous exposure to odorous substances, which causes manifestations in various organs.

MCS is a frequent disorder. According to the Canadian Community Health Survey, in 2003, 2.4% of Canadians aged 12 or older reported a medical diagnosis of MCS, much more frequently among women [1]. A more recent Canadian survey showed a prevalence of 2.85% among adults 15–80 years old [2]. In a U.S. survey in 2016, the medically diagnosed prevalence of MCS in the general population was 12.9%, compared with 3.9% in 2006 [3]. The same methodology was used in the two surveys, and the author did not address the factors that might explain this important increase in the prevalence of MCS in the USA. The prevalence is much higher when self-reported, varying from 6% to 33% according to surveys carried out in various countries, with varying degrees of severity [4]. Many factors may contribute to these wide variations, including different diagnostic criteria, different methodologies and sampling strategies, different populations and periods of study, different social influences, including negative perceptions about chemicals, and different socioeconomic statuses. Despite these variations, MCS remains a frequent disorder and a frequent comorbidity of chronic mental disorders, such as post-traumatic stress disorder, anxiety disorders, depression, somatoform disorders, and frequent comorbidity of physical chronic disorders qualified as medically unexplained syndromes, such as chronic fatigue syndrome, fibromyalgia, irritable bowel syndromes, and many others [5]. The prevalence is much higher in women, a constant feature, representing 69% to 88% of cases [6]. Veterans also have a high prevalence of MCS [7]. The disease is rare in children [8].

MCS has been renamed idiopathic environmental intolerance (IEI) since an international workshop in 1996 [9]. The term intolerance was preferred because no toxic or sensitivity/allergic mechanism had been established, and the term idiopathic was added because the origin remained unclear. The debate mainly opposed two visions: on one end, it would result from the effects of chemicals per se and their accumulation in the body by some toxic or biologic mechanism, and on the other end, it would result from a conscious or unconscious negative perception associated with chemical odors that trigger a stress reaction, or in other words, it is a psychosomatic disorder.

A vast number of symptoms (more than two hundred) and an almost unlimited list of associated chemicals have been reported among these patients [5,6,10,11]. For a given chemical, the manifestations may vary significantly between patients, and for a given patient, the manifestations may be similar for various types of chemicals. The usual medical tests performed on the target organs, as well as the toxicological tests, are generally normal. The diagnostic criteria have evolved over time, and a long list of treatment options has been proposed, but no evidenced-based treatment has been documented so far. Some do not even recognize the existence of this syndrome, given its subjective and variable manifestations, and despite its prevalence, the scientific evidence accumulated and the important impact on the life of the afflicted persons. All these factors contribute to the prevailing debate as to how this syndrome should be considered, classified, diagnosed, treated, and prevented.

Since the 2000s, much has been learned about its etiology and the underlying mechanisms, which allow us to propose a unifying theory and suggest treatment avenues. In this qualitative analysis, we aim to review the etiology and the underlying mechanisms of MCS derived from the scientific literature, apply this knowledge to typical cases of MCS stemming from a clinical practice in occupational and environmental medicine, and suggest treatment options that should be evaluated in research. Criteria for diagnosis and prevention strategies are also suggested.

## 2. Methods

The review of the scientific literature on the etiology and underlying mechanisms of MCS was mainly based on the systematic reviews, reports of expert groups, and meta-analyses already published, using the words MCS, IEI, environmental hypersensitivity, or environmental illness, mainly in PubMed. Among these, an extensive and systematic review published in 2021 by Carrier et al. from the Institut national de santé publique du Québec (INSPQ), which contains 840 pages and 4028 scientific articles searched from 29 databases, with a detailed analysis of the main studies, was an important source of information (full report in French, summary report in English) [5,12]. No other systematic review was attempted in this paper, but recent original studies not included in the previously mentioned documents were analyzed. Including the studies analyzed in the systematic reviews, the references span from the late 1950s to 2024 and include observational epidemiological studies, experimental studies on humans, systematic reviews, meta-analyses, and case reports. The results of the studies involving biomarkers and cerebral imaging techniques on MCS subjects are reported and compared with the results of the same types of techniques performed on subjects having the comorbidities frequently observed among MCS subjects.

The main etiological hypotheses derived from the literature were identified. An extensive analysis of all the etiological hypotheses proposed in the literature has been reviewed by Carrier et al. and was also reviewed in other expert groups and meta-analyses. The arguments for and against these hypotheses are synthesized. From this analysis, the main factors and mechanisms leading to MCS proposed so far are identified.

Based on the scientific literature and from the clinical experience of practice in occupational and environmental medicine with patients referred for MCS or IEI, a typology of MCS was looked for to identify distinct modalities or modes of presentation. No quantitative or statistical analysis was performed to identify these types, as these were suggested from the mechanisms identified from the reviews and evolved as the clinical cases referred matched the proposed mechanisms identified in the scientific literature. Cases illustrating each type were selected, considering the willingness of the patient to report her or his case. The patients have given their written consent for publication after reviewing the description of their case. The knowledge derived from the literature review was applied in these cases to explain the underlying mechanisms that may have contributed to the development of MCS and its effective resolution.

From all the data presented, a list of criteria is proposed for the diagnosis of MCS. Principles for effective treatment of MCS are suggested based on this analysis and from a review of the studies published on the effectiveness of trauma-focused psychotherapies. Recommendations are also made for prevention and therapies that could be evaluated in research.

## 3. Results

### 3.1. Etiology and Underlying Mechanisms

Despite the term “idiopathic” in IEI, most reviews in the last decades converge on the preponderant role of psychological and cognitive factors and stress-related mechanisms in the development of MCS. Various experts and scientific groups have reached this conclusion, therefore ruling out a toxicological or immunologic/allergic mechanism [5,6,10,12,13,14,15,16,17,18,19,20,21,22,23]. A brief summary of the main reports follows in chronological order.

In 1999, the American Academy of Allergy, Asthma and Immunology issued a position statement in which they mentioned that several medical societies and organizations had pointed out the lack of scientific support for and clinical evidence of the alleged toxic effects of environmental chemicals in MCS patients [13].

Staudenmayer et al. published two articles in 2003 in which they used Bradford Hill’s criteria of causality for reviewing the psychogenic origin of MCS and concluded that all the criteria were met and fulfilled those of a somatoform and functional somatic syndrome [21,22].

In a systematic review of provocation studies on MCS, published in 2007, Das-Munshi et al. concluded that persons with MCS do react to chemical challenges; however, these responses occur when they can discern differences between active and sham substances, suggesting that the mechanism of action is not specific to the chemical itself and might be related to expectations and prior beliefs [20]. The methods and the conclusions of this review were partly criticized by Goutsmit and Howes in 2008, pointing out that a group of these patients cannot identify a history of a significant event or a neutral stimulus they have experienced, in other words, a measurable conditioned stimulus may not always be present in the environment [24]. Consideration of the various factors and types of situations contributing to the development of MCS may suggest some explanations, as will be discussed further.

In the report of the Committee on Toxicity of Chemicals in Food, Consumer Products and the Environment, Statement on Idiopathic Environmental Intolerance, from United Kindom, published in 2011, the authors have concluded that we have found no convincing evidence for any biological mechanism that would explain why such diverse symptoms are induced in some individuals by such a wide range of chemicals, at levels of exposure well below those that are tolerated by the majority of people. Nor is there any convincing evidence of genetic differences in IEI patients that point to a toxic pathogenesis [6].

In 2017, Van den Bergh et al. proposed a comprehensive model for three types of IEI, including MCS [23]. They summarized a long list of laboratory studies that were performed on humans about symptom learning in response to chemicals. They stated that an extensive series of laboratory experiments have shown that, once a predictive association had been established between symptoms and a harmless but odorous chemical, those symptoms can be induced by presenting the odor alone. They state that a nocebo effect is implicated in IEI as the underlying phenomenon and that these individuals have a tendency to attribute symptoms to external causes and deny possible psychological explanations. Again, one may question if this documented mechanism (placebo effect) explains all the cases of MCS.

In their last UpToDate review about IEI (MCS), Black DW and Temple S state that many experiments and observational studies consistently identify psychopathology in patients with IEI and propose to consider MCS as a somatoform, depressive, or anxiety disorder [10]. They suggest psychotherapy as the primary option, based on what is recommended for somatoform and anxiety disorders. Medication is suggested as a secondary option.

The objective of the INSPQ report by Carrier et al., published in 2021, was to identify the pathophysiological mechanisms that underlie MCS by looking at all the research conducted on all the hypotheses put forward since the 1950s [5,12]. An important consideration was the strong association of MCS with related syndromes, such as chronic fatigue syndrome, electromagnetic hypersensitivity, fibromyalgia, post-traumatic stress disorder, chronic anxiety, depression, somatization disorder, phobias, and panic disorder. They report that in all the syndromes and pathologies studied, there was a disruption of the hypothalamic-pituitary-adrenal axis, an increase in inflammatory cytokines, a disruption in oxidative homeostasis, and a chronic decrease in neuromodulator levels (serotonin, dopamine, norepinephrine). In addition, using brain imaging, alterations in brain function and structure were observed that affect the limbic system circuits (emotions, memory, learning) and the prefrontal cortex (attention, reasoning, strategic thinking, judgment). As a consequence of these alterations, MCS-affected individuals develop neuronal sensitization. They conclude that chronic anxiety is an element common to all the syndromes studied, and a number of underlying factors may be involved, such as an individual’s temperament, personal history, and psychosocial makeup.

In her recent review (2023), KE Binkley mentioned that patients with IEI share many clinical features associated with anxiety disorders, as shown in experiments producing manifestations and pathophysiological responses typical of these disorders [18]. As she stated, the sense of smell is closely tied to the limbic system, arousal, and emotional memory, and odors are increasingly well recognized triggers of panic attacks, especially in individuals who have experienced a traumatic event. Among the factors leading to IEI, she identified notably childhood trauma, possible initial toxic exposure, current stressors, trauma, and various forms of learning (learned sensitivity, classical conditioning, symptom attribution, and social modeling). She made recommendations for the treatment based on these findings, mainly psychotherapy and, secondarily, pharmacotherapy.

At first glance, the following observations challenge the toxicological and allergic hypotheses [5,6,10,12,13,14,15,16,17,18,19,20,21,22,23]:

- Globally, there is an absence of specificity, as the symptoms for a given patient may be similar across various chemicals yet differ significantly among patients for the same chemical. Additionally, the number of implicated chemicals is innumerable, and the list of symptoms reported is very extensive. The symptoms can also be observed with other types of odors, such as sweat, feces, decaying or musty odors, and other organic odors;
- The dose that triggers the reaction is well below the recognized toxic effect threshold in terms of concentration and duration of exposure;
- The reaction caused by the perception of the odor is usually immediate, often within a second after the detection of the odor, although it may be delayed. For example, general symptoms can be triggered immediately following the perception of a scent, even before the substance has been absorbed systemically, therefore excluding a systemic toxic effect. We should mention that the chemicals cannot be transmitted to the brain by the olfactory structures [5,12]. A nerve impulse traveling in a fraction of a second from the receptors in the nose, through the olfactory nerve, and to the amygdala and the limbic system is a more plausible mechanism;
- The allergy tests generally show an absence of reaction to the various substances affecting these patients. Toxicology tests are also usually normal;
- The physical examination and usual laboratory tests related to the potentially affected organs are generally normal;

Notwithstanding these observations, research has clearly demonstrated that biological disturbances are observed among MCS patients based on studies using biomarkers and neuroimaging, as will be described further. The question is whether these are caused by the chemicals per se or by mechanisms underlying the associated disorders;
The following observations are in support of anxiety/stress/trauma-related mechanisms:

- Several studies have shown that these patients systematically have associated mental and physical chronic disorders, the onset of which most frequently precedes the development of chemical intolerance. These include the following: mental health problems such as somatoform disorder, post-traumatic stress disorder (PTSD), depression, generalized anxiety, panic disorder, personality disorder, and chronic physical disorders such as chronic fatigue syndrome, fibromyalgia, irritable bowel syndrome, tension headache and migraine, and food intolerances, to name a few [5,9,10,12,14,15,16,17,18]. A meta-analysis of 71 epidemiological studies with a control group has shown that the probability of developing chronic disorders such as chronic fatigue syndrome, fibromyalgia, irritable bowel syndrome, and others, as mentioned above, is closely related to a history of psychological trauma and particularly PTSD, in a dose–response fashion; i.e., the more a person has experienced traumatic events, particularly in childhood, the higher the probability that this person will be affected by one or more of these disorders [25,26,27,28]. The chronic fatigue syndrome is particularly associated with traumas and PTSD. In our clinical experience, a systematic and detailed interview of these patients has also shown the omnipresence of these comorbidities known to be associated with past emotional trauma and chronic stress;
- The high prevalence of associated intolerances (odors, noise, bright light, touch, etc.) of varying degrees cannot be explained only by chance [29]. Although in some circumstances, such as in certain workplaces, it may reflect a simultaneous exposure to various occupational risk factors (chemicals, noise, dust, heat), it most frequently indicates hyperarousal or hypervigilance. This response is a pathophysiological reaction of the autonomous nervous system that occurs following a traumatic experience to some form of physical or psychological threat and may be viewed as a way to quickly alert the individual of the presence of the same potential threat. It may develop early in childhood following psychological trauma(s) and is typically observed in post-traumatic stress disorder [30]. It may involve all the senses or some predominantly;
- In a systematic review of provocation studies on subjects with MCS, the authors have concluded that the onset of symptoms by chemical odors can be induced by cognitive and emotional factors [20]. Studies by Van der Bergh et al. have also shown that symptoms can even be triggered among normal subjects when they are exposed to innocuous odors following information on their potential toxicity [23]. As stated by Pitron et al., there is convincing evidence that expectancy induction can cause the acquisition of symptoms in response to chemicals unrelated to the chemical effects themselves, and that these effects are moderated by factors that also characterize MCS patients [31];
- In a high proportion of patients with MCS who volunteered for laboratory experiments, symptoms and physiological reactions typical of a panic attack or an anxiety reaction with hyperventilation were demonstrated [32,33,34];
- This syndrome has many similarities with the classical model of conditioning, more specifically, aversive conditioning [35,36]. Simply stated, when an innocuous stimulus, such as an innocuous odor, is repetitively associated with an independent health effect occurring in a stressful or threatening environment, this initially innocuous stimulus can eventually trigger the same health effect. This process is also called associative learning;
- Although these patients generally report a high olfactory acuity, their olfactory functions in terms of threshold, discrimination, and identification in controlled studies turn out to be similar to those of controls [20,37,38]. These apparently contradictory facts suggest that somehow, in the brain, not in the nose, their alert threshold to odors has been modified, probably in response to previous traumatic experiences, anxiety, and apprehension in order to more quickly alert of a potential threat;
- Genetic and metabolic abnormalities have been reported to be more prevalent among these patients, but this has been refuted by others [5,12,38,39,40,41]. Carrier et al. have systematically evaluated the studies looking at genetic susceptibility and have rejected this hypothesis using the Bradford Hill criteria of causality [5,12]. It should be mentioned that metabolic abnormalities are associated with chronic fatigue syndrome, a frequent comorbidity [42].

Carrier et al. reported the results of Luca (2010) and Dantoft (2014), who showed elevated blood concentrations of six types of cytokines in subjects with MCS, suggesting a chronic low-grade inflammation [5,12]. They pointed out that these results are similar to those of subjects with chronic fatigue syndrome and fibromyalgia, for example. They show that an immune response and a sterile inflammatory reaction are now recognized to be associated with psychological stress and neuropsychological or psychiatric problems. A psychological stressor can trigger a sterile inflammation of the central nervous system and a systemic inflammation. In addition, unresolved psychological trauma can induce persistent oxidative stress in brain cells, which disrupts cell function and energy production in the mitochondria (which may explain the symptoms and manifestations associated with mental and physical fatigue), accelerates cell death, and is associated with a higher risk of various neurodegenerative disorders such as Alzheimer’s disease [42,43,44,45,46].

Carrier et al. reported studies that demonstrated that chronic oxidative stress leads to a reduction in the concentration of the following neurotransmitters: serotonin, noradrenalin, adrenaline, and melatonin [5,12]. This phenomenon could explain all the symptoms reported by the MCS subjects (psychological distress, concentration difficulties, memory loss, chronic fatigue, sleep disorders, etc.) and is also observed in all medically unexplained disorders and all the MCS comorbidities.

Brain imaging studies have been performed among patients with chemical intolerance and have been reviewed by Rossi et al. and Carrier et al. [5,12,47]. Carrier et al. provide an extensive description and analysis of these studies, which will be the focus of this summary. Some studies of particular interest are summarized.

The study by Orriols et al. involved eight subjects with MCS and as many controls challenged in the presence of non-toxic chemical odors under experimental conditions [48]. Psychometric and brain imaging tests (SPECT) were performed before and after the challenge. A perfusion problem was demonstrated in specific areas of the brain before and after the challenge in the eight patients but not in the controls. The authors state that their study showed brain SPECT dysfunction, particularly in those areas involved in odor-processing, when patients experienced the symptoms of MCS after chemical exposure, and suggest a neurologic pathogenesis of this disorder rather than psychogenic. But these observations are coherent with the role that these structures play in the brain, as parts of the olfactory system, in the detection and reaction to threat, and in memory and emotions [49]. Carrier et al. pointed out that the chemical substances used in this study were those familiar to the MCS subjects so that the exposures elicited unpleasant memories that provoked strong emotions and neurological reactions associated with avoidance behavior. This may explain why neocortical structures inhibit sensory signals in the limbic structures of the olfactory system [5,12].

Hillert et al. performed an experimental study on 12 MCS subjects and 11 controls, aged 22–44, all working or studying females, who were subjected to a PET technique where 5-HT_1A_ receptor binding potential (BP) was assessed after bolus injection of a radioactive marker [50]. In humans, studies have confirmed that the binding affinity of the 5-HT_1A_ receptor, which is linked to the serotonin neurotransmitter, is reduced in conditions associated with avoidance, such as anxiety and depression, which are comorbidities of MCS. The study showed that these MCS subjects had prior increased harm avoidance and anxiety. The tests showed specific reductions in the 5-HT_1A_ receptor BP in the amygdala, the anterior cingulate cortex, and the insular cortex. The authors state that the results imply that changes in the serotonin system may provide a physiological ground for the increase in harm avoidance and, perhaps, in top-down modulation of the response to odor stimuli. These observations suggest that the top-down modulation results from their anxiety and harm avoidance behavior, which are probably linked to prior stressful or traumatic events.

In a recent publication by Molot et al., the authors propose that the development of MCS may be attributed to the sensitization of transient receptor potential (TRP) receptors, notably TRPV1 and TRPA1 [51]. They state that MCS has often been inappropriately viewed as stemming exclusively from psychological disturbances and propose that greater recognition of receptor-mediated biological mechanisms should be incorporated into laws and regulations. In response to their article, a group of authors from diverse universities around the world argue that this view is not scientifically valid, that correlation is not causality, and that it maintains the opposition between the biological and psychological origin of MCS [31]. They rather propose to adopt a biopsychosocial model, as suggested by WHO. Unfortunately, the presence of objective signs of alterations in the brain is frequently interpreted as excluding the implication of psychological factors.

In 2015, Belpomme et al. published a descriptive study of consecutive patients who consulted at the same clinic over the years and involved subjects with intolerance to electromagnetic fields, chemical odors, or both [52]. Biochemical and brain imaging abnormalities were observed in variable proportions of the subjects, suggesting an inflammatory process in the brain and hypo-perfusion in the limbic system. Carrier et al. have criticized this study and found errors in the reported units of blood markers (for instance, given the units of blood histamine indicated in the article, 40% of the patients would have had an anaphylactic reaction at the time of the test), errors in the interpretation of the encephaloscan (an imaging test only used in this hospital in France), and the absence of a control group [5]. In 2018, Irigaray et al. published the results of the ultrasonic cerebral tomosphygmography (or encephaloscan) reported in the prior study among 353 patients with electrohypersensitivity (EHS) (66%) and 182 with both EHS and MCS (34%), using a control group [53]. Relative to normal controls, the results showed a significant decrease in the mean tissue pulsometric index, predominantly in the capsulo-thalamic and adjacent areas, in more than 80% of the patients, suggesting some vascular and metabolic impairment. No data are available for MCS patients only. It is difficult to determine if the anomalies identified by Carrier et al. in the first article have been eliminated. The authors argue that the abnormalities observed are secondary to exposure to chemicals or electromagnetic fields and deny any influence of psychosomatic origin. No consideration was given to the comorbidities, in particular to anxiety, stress-related disorders, and past emotional trauma, as these variables were not controlled in the analysis.

Using standard imaging methods (which differ from the one used by Belpomme et al.), studies have shown wide documentation of brain imaging abnormalities associated with post-traumatic stress disorder (PTSD), emotional traumas, and other associated mental disorders, and documentation of biological markers of oxidation and inflammation associated with these disorders as previously mentioned. Several systematic reviews and meta-analyses have demonstrated among these subjects, particularly those with post-traumatic stress disorder, the presence in brain imaging of functional and structural abnormalities, including a decrease in vascularization and atrophy in certain brain areas [54,55,56,57,58,59]. The results of a large-scale neuroimaging consortium study on PTSD have demonstrated that PTSD is associated with smaller hippocampus and possibly amygdala volume, both structures having ample a priori evidence implicating their role in PTSD [60]. There is animal experimental evidence showing that the decrease in vascularization and atrophy in these brain structures may be reproduced under repeated threats and is related to the cortisol level. There are also data in human studies corroborating this mechanism. Recent clinical studies have also shown that the structural and functional alterations observed in brain imaging (using various techniques) among PTSD patients disappear after effective psychotherapy, mainly Eye Movement Desensitization and Reprocessing (EMDR) and trauma-focused Cognitive Behavioral Therapy (CBT) [61]. The authors conclude that these studies provide neurobiological evidence of the role played by the prefrontal and limbic structures in patients with PTSD and the effects successful psychotherapy may have on these structures.

Considering the extent of the scientific evidence previously mentioned, namely the high prevalence of anxiety traits and stress/trauma-related disorders among the subjects with MCS, considering the known effects of psychological trauma and anxiety on the structure and functioning of specific brain structures, considering the disappearance of these brain abnormalities in brain imaging following a trauma focus psychotherapy, it seems likely that the observed effects, for instance in the study of Belpomme et al., result from unresolved psychological trauma and anxiety and stress-related disorders rather than from exposure to very low doses of chemical substances.

Many factors are probably involved in the development of MCS, including anxiety, psychological traumas, periods of high stress and threat, prior physical and mental diseases, occupational and environmental exposures, familial and social influences, erroneous interpretations and beliefs, and innate psychological and cognitive mechanisms as well as physiological responses of the autonomous nervous system, which can all interact in a complex way in the development of the syndrome. But among these, psychological and cognitive factors play an important role, and if these are properly taken into account, they support the view that MCS can be treated and should not be considered a permanent disability.

### 3.2. Typology of MCS

Given the wide manifestations of MCS and the various modes of presentation, we looked for a typology that would structure these variations and help to identify the etiological factors and the underlying mechanisms for every case, which can guide the choice of an effective treatment. From the scientific literature and the main factors and mechanisms associated with the development of MCS, we identified the modes of presentation that seemed distinct and common. These factors were summarized by Binkley as childhood trauma, possible initial toxic exposure, current stressors, and trauma, as well as various forms of learning [18].

This qualitative analysis has led to the identification of three common types of presentation of MCS, which can coexist: (1) following an initial toxic exposure causing an associated emotional trauma, called the accidental type; (2) following a repeated innocuous exposure in a stressful and threatening context or environment, called the associative type; (3) and following a hypervigilance acquired at a young age, secondary to repetitive threats, which has later evolved to a more pronounced chemical intolerance, with other environmental intolerances and other stress/trauma-related disorders, called the developmental type. Note that some mechanisms may overlap these types.

#### 3.2.1. The Accidental Type

This was the first type identified in the occupational setting and corresponds to an initial description of chemical hypersensitivity following an initial toxic exposure by the physician M. R. Cullen [62]. It also corresponds to the cases reported by Shusterman [63]. The following case illustrates this type of MCS.

##### First Case

During winter, in January, a man working for the same company for 40 years complained of a bad smell in his truck associated with a general malaise, a feeling of a lump in the stomach, shortness of breath, dizziness, nausea, and much fatigue. The symptoms gradually subsided at home, but the severe fatigue persisted. The next morning, he used a carbon monoxide detector to check his truck; the detector sounded the alarm in the driver’s cabin and in the back cabin. He had an immediate mild headache and nausea. He then shut down the engine, the heater, and the generator, all fueled by gasoline, and ventilated the truck. In the afternoon and evening, he had urgent work to do while the truck’s generator and heater were shut down. At the end of the day, he had the same symptoms. At around midnight, he contacted the poison control center and was referred to the hospital. The emergency physician diagnosed a carbon monoxide (CO) poisoning (carboxyhemoglobin level initially at 2.7%; a non-smoker) and told him he could have died if he had not acted promptly. He was treated with normobaric oxygen for about 4 h and became asymptomatic. He returned home and slept most of the day. Some days later, he and a health and safety coworker performed tests that showed a problem with CO in his truck. He informed his employer and used another truck while observing some persistent symptoms. Two weeks later, tests performed by the employer in his absence showed no problem with CO in his truck. He was forced to use the same truck, contrary to the advice of an occupational physician. In mid-February, he had to stop work following a shoulder injury. His symptoms slowly subsided at home. He returned to work in May. He was forced to use the same truck without any modification. He objected without success. His compensation claim was contested by the employer. Around May or June, he began to react to various odors, including gasoline, cigarette smoke, perfume, and sweat. These smells triggered an immediate reaction: a feeling of a lump in the stomach, tightness in the chest, and fatigue. He had to wear a cartridge mask to put on gasoline in the truck. Just seeing or hearing a neighbor’s vehicle start up across the street immediately triggered the same symptoms. In addition, he became intolerant to bright light, such as vehicle LEDs, which dazzled him. He used a CO detector a few times while running the truck. On approaching a truck, the detector could indicate a slight concentration (less than 10 ppm), which immediately triggered his symptoms, then return quickly around zero when moving away. In January, one year after the initial event, he was allowed to perform telework and modified tasks. At home, he complained of occasional chest tightness, persistent fatigue, and recent memory problems.

Beginning in March (more than one year after the initial event), he was treated with Eye Movement Desensitization and Reprocessing (EMDR) psychotherapy. The main events associated with his workplace were the focus of treatment, as well as some prior personal traumatic events. His physical symptoms gradually subsided, and the exposure to odors produced no symptoms in most circumstances but few symptoms for a brief moment in some others.

This first case illustrates the development of an MCS following an initial toxic chemical exposure associated with some odor, which was first notified as an occupational poisoning but was later contested by the employer, provoking a conflictual and persistent threatening situation. This man was treated for CO poisoning and was told by the physician that he could have died if he had not acted properly, which was recorded in his memory as a serious event. Following contrary data, the employer contested the initial diagnosis and his claim and rejected the preventive measures he asked for. This has provoked a persistent sense of threat and the perception of a lack of respect and consideration for his professional experience. The initial event was associated with emotional trauma because of his perception of potentially lethal consequences, but this was maintained and amplified in the following months as the perception of threat and negligence were maintained. Chemical intolerance started to develop during this period. Intolerance to the odor of gasoline worsened quickly, and even the sound of an engine could trigger the same symptoms. Note that the symptoms triggered by the odor of gasoline were similar to those of the initial event. Erroneous beliefs, i.e., misunderstanding of the conditions necessary to cause CO poisoning, may have contributed to a perception of threat while he was driving and coming close to a truck. The repeated experience of immediate physical symptoms triggered by these situations may have reinforced his perception and reaction. The development of intolerance to bright light may indicate an association with truck lights in the initial event or a result of general hypervigilance.

This case resembles that of PTSD in an event where an odorant chemical (gasoline) has caused physical damage with an associated emotional trauma. It also resembles the initial description of chemical hypersensitivity made by Cullen [62]. Shusterman et al. have described two similar cases that occurred following an acute toxic exposure and reported that their reaction disappeared following early explanation and reassurance as a consequence of hyperventilation [63]. In a case-controlled study, Pages et al. have reported cases of PTSD associated with CO exposure following a storm [64]. As other examples, we have treated workers who developed MCS after they were poisoned by acute and chronic exposure to organic solvents, who faced opposition from the employer to take the actions needed to control the exposure, and who denied their occupational disease.

The intolerance to the odor of gasoline in the present case has quickly generalized to a few chemical odors, as well as sweat. In other instances, it could generalize more or less rapidly to multiple related and unrelated chemical odors, which is explained by a learning mechanism, as the brain naturally seeks to generalize during the acquisition of knowledge, especially in the case of a threat [36]. The physical damage associated with the initial traumatic event does not need to be severe, but it must be interpreted as serious by the patient, as the perception of a threat is an important factor in the development of trauma and post-traumatic stress disorder. Prior personal events and anxiety traits, in this case, may have contributed to his increased perception of threat. The main traumatic events were processed with EMDR psychotherapy, an evidence-based psychotherapy for PTSD, and the odors were considered a trigger of the traumatic memory and treated as such according to EMDR protocol [65].

In the case of an accident involving multiple exposures in the workplace (noise, heat, fire, dust, chemical), the development of multiple intolerances could be explained by a specific association with all these stimuli. In many instances, though, a hypervigilance affecting many senses is expected, whatever the nature of the event, as in PTSD.

In other instances, the patient may not be aware of the presence of an odor in the initial traumatic event. During such an event, the information perceived by the senses can be recorded in the memory without the conscience of the person whose attention is entirely captured by the threat. But it may be memorized and used in the future by the autonomous nervous system as a quick alert, which will provoke a stress response with the same negative thoughts, emotions, and sensations as those experienced during the initial traumatic event. It should be mentioned that olfactory memory is especially known to be closely associated with emotions, positive or negative, as in PTSD, and may be particularly persistent [49]. Any type of odor, chemical or biological, which is associated with a frightening and traumatic event can trigger a stress reaction when re-exposed to a similar odor. An example would be the development of intolerance to the odor of alcohol and tobacco smoke of a woman following an assault by a man giving off these smells. The same mechanism could explain the reported association of MCS developing among women following unexpected emergency caesarian [66,67] or among patients admitted unexpectedly to the intensive care unit in a serious condition, for instance, with a severe COVID-19 infection. In these situations, the type of presentation is traumatic and accidental, but it also shares one feature of the associative type, in that the chemical exposure they have experienced was not toxic, contrary to those cases described above.

The development of an excessive intolerance to heat and humidity following a threatening heat exhaustion or heat stroke is an example of an environmental intolerance caused by a physical agent and an associated psychological trauma. The development of intolerance to electronic devices or electrohypersensitivity among those who have experienced a serious electrical burn could possibly be explained by the same mechanism. A thorough discussion of electrohypersensitivity, which shares similar as well as distinct features with MCS, is beyond the scope of this article.

#### 3.2.2. The Associative Type

A second type of MCS was identified in the occupational setting and was frequently observed: the development of an intolerance to an initially harmless odor, which later evolved as a potent trigger in a conflictual and stressful context and problematic environment. The word associative refers to the process of associative learning, a synonym of aversive conditioning, and refers to the repetitive exposure to an innocuous chemical exposure while some threatening factor is present in the same period or environment.

##### Second Case

About one month after starting to work in a school in 2014, a female teacher in her 50s developed various symptoms, including itchy and burning eyes with morning secretions, nasal secretions, sometimes greenish, nasal congestion, headaches in the forehead, pain and blockage in the ears, dizziness, loss of voice, difficulty breathing, and much fatigue. Her symptoms were reduced on days off and increased on return to work. Diagnoses of conjunctivitis, rhinosinusitis, vestibulitis, and asthma were established, all caused by fungal contamination of her workplace. Indeed, the history of the building revealed that recurring water infiltrations had been present for years, and the tests carried out showed contamination by molds, particularly important in the basement where she worked. Colleagues complained of similar symptoms associated with their presence in the school.

Her condition worsened despite medications targeting all her diseases. She was removed from work at that time. Her condition slowly improved, and the physical examination also normalized over a period of a few months. She was able to stop the medications.

She returned to work in July 2015 at another school. At that time, the building was undergoing major work affecting the roof and the exterior brickwork due to water infiltrations. When she returned from vacation in August, her symptoms of conjunctivitis and rhinitis reappeared. She asked to be relocated to another floor, away from the work, which was agreed upon and greatly reduced her symptoms.

As of September 2016, she was assigned part-time in the same school building and part-time at another school. Each time she worked in the latter, she had headaches and dizziness, as well as nasal congestion and posterior secretions, with earache and tinnitus. The physical examination was abnormal and corroborated her symptoms. Two other colleagues had symptoms associated with their presence at work. This building was old and had undergone recent work. A relocation recommendation was made but was denied. Negotiations with the employer were increasingly difficult, and her health problems related to the workplace were not recognized.

Beginning in the fall of 2016, she noted an intolerance to the odors of the detergent used to clean the school floor once a week, which quickly caused a tightness in her throat, difficulty breathing, and dizziness, requiring her to go outside. She felt more and more affected by her work environment and felt abnormal or inferior compared to other colleagues. She feared being fired because of her reactions and was anxious about her condition.

In March 2017, she was removed from the workplace because of her health problems. Her symptoms completely resolved in the following months. She was then considered suited for a return to work in a sanitary building, which was not found or proposed.

In March 2018, she seemed increasingly affected by products that give off a strong smell, such as glue and silicone, which immediately caused her to feel dizzy with breathing difficulties. She was anxious and tormented due to the pressure she was under to return to work, with a difficult choice, as she loved teaching but dreaded being sick again or wondered if she should retire early.

She was referred to a psychotherapist. A particularly disturbing event that she experienced at work was targeted and treated with the Flash technique: while she could no longer go upstairs, she was choking and exhausted. At the start of the Flash technique, she experienced difficulty breathing, similar to that experienced at work during this event. This difficulty then gradually subsided during the first session. At the second session, she no longer felt any distress related to this event. Then, she was desensitized to the perception of an odor by the same technique, thinking about what she felt at that moment. Subsequently, at home, she briefly exposed herself to a similar odor using the same technique and repeatedly observed the lack of reaction. She never experienced any odor-related symptoms again. Moreover, she no longer perceived herself as inferior but different from the others.

In this case, the patient developed an MCS following the repetitive occurrence of serious health effects caused by her work environment within a context of neglect and denial of her occupational diseases and consequent high stress. This was associated with repetitive exposure to an innocuous and odorous chemical in her classroom, which, for many years, was not causing any symptoms. The MCS generalized to some other chemical odors following increased stress and conflict related to the pressure she had to return to work in unsanitary conditions. Despite the strong evidence that her many diseases were caused by repetitive water infiltrations and molds, which all resolved following her retrieval from the workplace and medications, the employer has contested the occupational claim without the opportunity for her to be relocated. The repetitive association of physical symptoms in a stressful environment and exposure to innocuous chemical odors in her classroom have produced aversive conditioning so that the innocuous odor triggered an immediate stress response.

Water damage and molds are highly prevalent in various types of buildings, and there is ample scientific evidence that these can cause a variety of respiratory diseases and others, such as those experienced by this woman [68,69,70,71,72]. The sick building syndrome and MCS have also been associated with dampness and molds [73,74]. Besides the objective disorders mentioned above, the entity called dampness and mold hypersensitivity syndrome (DMHS) has been proposed for those patients who react immediately to a damp or moldy environment without objective clinical alterations [74,75]. If mold exposure is causing a serious disease, and particularly if it is firmly contested, this may create a threatening situation, facilitating the unconscious association with odors and other factors in the immediate environment, as in this case. But in any stressful situation related to the workplace or other environment, such as harassment, fear of losing a job, or conflict with an authority or a relative, MCS is susceptible to develop, even if the person has no deleterious effect of a toxic, allergic, or infectious nature caused by the indoor air or the indoor environment. This is more susceptible to occur, for instance, for a person with an anxiety disorder. Given the frequency of such problems in the workplace, this type of MCS is frequent. The association could occur in various circumstances associated with the workplace or not, such as a health professional developing an intolerance to a disinfectant associated with high stress in the workplace, an intolerance to some odor in the living apartment following a conflict with the neighbors, or an intolerance to an odorous cream for skin disease in a period of high stress.

Discerning a toxic or allergic reaction from a stress reaction may sometimes be difficult. For instance, in this case, if the symptoms are triggered instantaneously following the perception of a musty odor or simply entering a specific room, and if these do not match the known health effects of molds, then a stress reaction may be suspected, given these typical manifestations. The history should reveal the stressful circumstances associated with this period and environment. But if a reaction congruent with the known health effects of molds appears in the following hour or so, it suggests a mold reaction, and objective signs may be observed if the patient can be examined shortly after. Both types of reactions can also occur in the same person.

#### 3.2.3. The Developmental Type

Many patients have been referred for an MCS for which exposure in the occupational setting was not raised but started at the developmental period and evolved in adult life to a prominent intolerance leading to many restrictions in their life. Staudenmayer et al. had already suggested in the study published in 1993 that childhood trauma was associated with the development of MCS [76]. A prominent feature of these patients was the presence of many comorbidities known to be associated with chronic stress and past emotional trauma in the period of development.

##### Third Case

A woman in her 40s has been sensitive to various chemical odors for the past 12 years, including exhaust fume and smoke (from cigarettes, BBQs, and campfires), which instantly cause nasal symptoms and chest tightness, while the smell of gasoline triggers immediate nausea, and the smell of bleach causes irritation of the nose, throat, and airways, chest tightness, and asthma attack. She is also intolerant to noise, bright light, vibrations, and touch. She also suffers from obstructive sleep apnea, chronic fatigue, fibromyalgia, anxiety, and celiac disease. For years, she had been very much affected by various respiratory diseases (rhinosinusitis, asthma, repetitive respiratory infections) caused by mold contamination in her house; these illnesses gradually resolved after leaving the residence.

On a summer day, following an application of an herbicide on the lawn of her residence, redness and itchy skin appeared as soon as she perceived the odor in the house. The next day, she experienced chest pain, dizziness, fatigue, and great anxiety. She could perceive the odor of the herbicide for a few days.

A review of her personal history has revealed that she had suffered multiple unresolved psychological traumas since childhood. As a child, she suffered the ever-present threat of physical punishment from a babysitter (of which she has few memories) and from her mother. As a teenager, she suffered bullying and had an auto accident, causing injury and untreated PTSD. In adulthood, she developed various incapacitating physical illnesses in a contaminated house, had to accumulate many tasks despite her severe fatigue and pain, and experienced significant difficulties with a manipulative and narcissistic spouse.

Her many traumas were treated over a long period with EMDR psychotherapy by an experienced psychologist. She regularly used a self-administered desensitization program, meditated, read related books, and did breathing exercises regularly, all of which helped to improve her general condition and reduced her fatigue, as well as her general pain and odor intolerance. Later, she experienced increased fatigue following the occurrence of financial difficulties and lengthy stressful legal procedures in connection with her ex-spouse.

In this case, MCS represents an evolution over many years of hypervigilance, which started early in childhood. The repetitive threat of punishment from her mother and the babysitter in the early years of development is an important trauma that has built the basis of her hypervigilance, as shown by her early reactions to many environmental triggers. Her memory is unclear about the possible exposure to bleach from the babysitter in her first years of life. Other important traumas in adolescence and adulthood have contributed to maintaining or increasing her hypervigilance, as well as to the development of comorbidities such as general anxiety, chronic fatigue, fibromyalgia, and sleep apnea [77]. Exposure to molds with significant physical consequences in a period of persistent stress may have contributed to a further increase in her intolerance. Other contributing factors include general anxiety and some erroneous beliefs about chemicals. Diverse therapies were used, including meditation, breathing exercises, mental exercises, cognitive restructuring (from therapists and lectures to understand the basis of her diseases and the distinction between toxic exposure and stress reactions), and EMDR psychotherapy targeting her multiple traumas, all contributing to improving her health status.

As previously mentioned, the link between MCS in adult life and childhood physical or sexual abuse was suggested in a case–control study published by Staudenmayer et al. [76]. In our practice, this type of MCS seems quite frequent, which reflects the high prevalence of abuse, neglect, and violence, particularly in childhood and among females. For instance, in a recent survey conducted in Quebec, a region not known to be particularly violent, almost half (48%) of the children were victims of psychological aggression repeatedly (three times or more), and 3.4% of children experienced at least one episode of severe physical violence, during the 12 months preceding the survey [78]. In the USA, the best studies provided an estimated lifetime prevalence of PTSD among American civilians of 6.1%, but 4.1% among men and 8.0% among women [79]. Women are also much more frequently victims of sexual violence, and only women experience delivery. Therefore, it is not surprising that MCS and other associated stress/trauma disorders are more prevalent among women.

In this mode of presentation, there is frequently an absence of memory of a specific exposure to chemicals, which may provide an explanation as to the absence of an unconditioned stimulus in a group of MCS cases, as previously mentioned by Goutsmit and Howes [24].

Hypervigilance is a pathophysiologic response that occurs following one or multiple experiences perceived as a threat in some way. The threat may relate to a wide range of situations of varying severity: the event may be dramatic, such as incest, a rape, an assault, or a violent accident, or result from repeated exposure to situations such as denigration and negligence during development, or psychological harassment [26,30]. The event may have occurred a long time ago or recently, be single or repetitive, and the nature of the threat may be physical or psychological, or both. In the short term, hypervigilance can be viewed as an adaptive and protective mechanism aimed to quickly alert a person of a danger of the same kind. But in the long term, its maintenance becomes maladaptive and deleterious as it triggers an uncontrolled stress reaction in situations where there is no real threat. In a threatening situation, the memory records the signals detected by the senses, most often without the awareness of the individual. When something in the environment similar to the threatening event is detected, it triggers an immediate stress reaction that the person may not understand. The hypervigilance may involve not only the sense of smell but also the perception of sounds, light, touch, vibration, and heat. All these senses are connected to the amygdala in the brain, which is responsible for the surveillance of potential threats in the surrounding environment. It may not be possible to find a specific odor associated with the initial trauma, as the hypervigilance may impact all the senses. This physiologic phenomenon can evolve to a more or less severe intolerance in adult life, for instance, following exposure to an odorant chemical in a period of stress exhausting the adaptation capacity, as in the present case.

Some patients report that they can react to a chemical before they perceive its odor. Various mechanisms could be involved. The mere presence in a place or a room with visual cues associated with past chemical exposure and trauma may be sufficient to trigger a stress response, given that studies have shown that olfactory and spatial memories are closely related in humans [80]. It could be explained by a systematic search for a specific exposure and specific attribution following a reaction, as many of these patients tend to systematically look for the same exposure and explanation for their symptoms. It can also be attributable to a very low odor threshold, which has been constantly reduced by re-experience; indeed, as the nose can differentiate various odors by their molecular specificity, this suggests that the reaction to chemical molecules could precede the perception of its odor when the threshold has become very low.

### 3.3. Criteria of Diagnosis

Many organizations have underlined the absence of a standardized method of diagnosis and treatment of MCS [11]. The wide variety of its manifestations and the absence of consensus on the etiology may partly explain this fact. So far, no objective marker has been proposed for the clinical diagnosis. Based on the characteristics observed among this typology of cases and the underlying mechanisms derived from the scientific literature, the following diagnostic criteria are proposed. Except for the first one, not all the following criteria are essential for the diagnosis of MCS, but a majority should be met.

- Exposure to odors at a dose not considered toxic

Exposure to odors at a dose not considered toxic is the fundamental criterion. This should not be confused with a history of prior accidental toxic exposure at work in some cases. It may not be easy for someone unfamiliar with toxicology to determine if the dose is toxic, but most of the time, it is quite obvious. For instance, in the first case, a toxic dose of CO is suggestive in the initial event, but when starting to fill the truck with gasoline or when observing a brief and slight increase in CO concentration while driving, these are exposures obviously not toxic and do not deserve toxicological test. The credibility of the health professional and the time and care this professional has taken to exclude a toxicological mechanism are important for the patient to accept that he has MCS, not poisoning.

- Low odor threshold

The majority of these patients report a high olfactory acuity, some describing their nose as bionic, while the odor may be considered slight or even undetectable by relatives. This phenomenon may be present since childhood or follow specific events in adulthood. This occurs because of a harm avoidance behavior programmed in the brain, not a dysfunction in the nose, as previously explained.

- Rapidity of occurrence of symptoms following exposure

Following the detection of the odor, for instance, a perfume, a common detergent, tobacco smoke, an undefined smell, or an organic smell, the symptoms are usually triggered almost instantaneously, but sometimes not rapidly. The symptoms may disappear rapidly following retrieval of exposure, but some symptoms, such as fatigue and confusion, may persist for hours or some days, which are the result of acute stress reaction over pre-existing chronic stress.

- Generalization to related and unrelated chemicals

The reaction may first be associated with one or few chemicals, but as the syndrome evolves, the reaction may generalize more or less rapidly to a variety of related or unrelated chemicals, be quicker and more severe. In some cases, the intolerance remains related to one dominant odor or some odors, while in other cases, it can include an almost unlimited list so that the number of chemicals to which a person is intolerant should not be a criterion of diagnosis.

- Non-specificity of symptoms

The list of symptoms reported is extensive (a few hundred), so the manifestations vary greatly and do not necessarily match the known toxicological properties of the substance. It frequently affects the upper and lower respiratory tract, the central and peripheral nervous system, the circulatory system, the digestive system, and the skin, but all the systems may be involved. For instance, the patient may report an immediate sensation of irritation (tingling, burning) in the nose and throat, cough, and respiratory distress, followed or not by other symptoms such as nausea, headache, irritability, mental confusion (brain fog), sudden fatigue, lightheadedness, palpitations, sweating, red skin, some of which may be persistent. For a given patient, the symptoms may be similar for diverse chemicals, and for a given chemical, the symptoms may vary between patients. The symptoms may be similar to those experienced in the traumatic events if these can be identified. Chronic symptoms are confounded by those of the comorbidities and can all be triggered or increased by the perception of odors. As Carrier et al. have pointed out, the chronic symptoms are related to chronic neuroinflammation, endocrine disruption, decreased neurotransmitters, and oxidative stress, which cause notably altered mood and decreased motivation, sleep disturbances, fatigue, and decreased energy [5,12].

- Similarity of symptoms to those of acute stress reaction, panic attack, or hyperventilation

As previously mentioned, studies have shown that a high proportion of patients who volunteered for laboratory experiments developed symptoms and physiological reactions typical of a panic attack or an anxiety reaction with hyperventilation [18,32,33,34]. The description of sudden breathing difficulties, palpitations, and faintness, for instance, suggests an acute stress response such as a panic attack or hyperventilation. The persistent mental and physical fatigue is another clue to an acute stress reaction.

- Normal physical examination and normal usual tests in the target organs, and no other explanation for the reactions

Other potential causes should be assessed before concluding MCS, but care should be taken not to order endless tests, as the patient may be pressing to do. The physical examination and the usual medical tests in the target organs, as well as toxicology tests, are generally normal, but in laboratory experiments and research, various alterations have been noted, as previously mentioned. There is no biologic marker that has been accepted so far and could be applied in a clinical setting. We should not forget that a patient may have more than one disease at the same time. For instance, an allergy may be documented to some allergens causing nasal symptoms, but most chemicals to which these persons react are not allergenic. Another example would be someone who had respiratory diseases caused by an indoor air problem and who also developed an MCS related to aversive conditioning, as in the second case presented.

- Intolerance affecting other sense(s)

As shown by Palmquist et al., other forms of intolerance are frequently associated, which can manifest as an immediate and exaggerated reaction to noise or sounds, bright light (neon, sun, LED), touch, vibration, heat, or electric/electronic devices [29]. The intensity of the reaction may vary according to each sense, and the patient may only report a sense of malaise, aversion, or threat by these triggers rather than specific symptoms. This generalized intolerance is not always present but is most frequently seen in patients who have untreated or chronic PTSD or among those who have developed hypervigilance early in their life following repeated traumas. It can also be seen in the case of multiple simultaneous types of exposure in the workplace.

- Erroneous beliefs about chemicals or odors

Erroneous beliefs about chemicals may be present for a long time or recently and be in general or only related to specific chemicals. The person may be searching for explanations and find on the web or from relative’s negative opinions and generalizations about chemicals without considering the dose involved, which may initiate or reinforce their beliefs about chemicals. For instance, an odorous essential oil may not be considered a chemical and, therefore, not toxic, contrary to a very brief exposure to a chemical perfume. They may focus on the systematic identification of chemicals as the cause of their symptoms, neglecting the contribution of stress and other factors. Cognitive factors may interact at different points during the process of MCS and should be dealt with accordingly.

- Comorbidities, physical and psychological, associated with trauma and stress

Comorbidities are frequent and have been mentioned, such as chronic fatigue syndrome, fibromyalgia, irritable bowel syndrome, migraines, and other physical disorders, as well as many psychiatric and somatoform disorders [5,10,12,14,15,16,17]. These should be part of the questionnaire.

- History of unresolved emotional traumas

A history of past emotional trauma should be systematically looked for, keeping in mind the wide variety of situations that may be traumatic and the reluctance of many patients to acknowledge them. This may be the most difficult part of the history, and the physician should mention at the beginning that a thorough questionnaire and examination will be performed, including personal life history beginning in infancy, and obtain consent to do so. It may take more than one visit to gain trust and obtain such personal information.

Many factors may preclude the identification of past exposure to some form of trauma. Patients may not remember events that occurred a long time ago. This is especially true in the case of dissociative amnesia, which particularly occurs when children are exposed to unbearable trauma, such as incest. A person may not report past events when she/he believes that they occurred a long time ago and should not impact her/his life anymore. The patient may have consulted a psychotherapist for months or years and therefore reject the impact of past traumas, but not all therapies and therapists are equally effective in this regard. The person may also be ashamed to report a traumatic event. In other cases, the person may intentionally not report such an event for various reasons, including the fear of negative perceptions from the health professional or the belief that their physical reaction cannot be psychosomatic. Indeed, the nature of true physical symptoms, their suddenness and uncontrolled features, without a conscious perception of threat, often convince the patient that this reaction is directly caused by a toxic or allergic effect of the chemical and cannot be psychogenic or explained by a stress reaction. They often perceive that a psychological root means that they are weak or faking, which is insulting. Some may persistently look for an external cause, physical or environmental, of their diseases and acquire accordingly strong beliefs about the toxic effects of chemicals in daily use. In all cases, good communication skills are important to obtain such information, including time, patience, non-judgmental listening, and trust. A written questionnaire is generally not sufficient to obtain a good history of past traumas. A disturbing memory should not be detailed before being treated so as not to revive the traumatic memory and not to worsen the patient’s condition.

### 3.4. Treatment

Few studies have been conducted to evaluate the effectiveness of treatments for MCS, and as mentioned in all reviews, there is actually no treatment for MCS that is evidence-based [11]. But many authors have recommended treatments based on those that are formally recommended for the comorbidities, which may partly explain why little research has been performed specifically to document the effectiveness of treatments for only MCS. Another factor is the reluctance of many patients to consider psychotherapy as they reject the proposed etiology.

It is interesting to note which treatments the patients have reported to be most effective, as reported in a large sample of MCS patients in the USA: avoidance of potential exposures, elimination of chemicals in their own environment, and prayer have been reported as the three most effective, among a long list of strategies and therapies [81]. Recommendations based exclusively on the strict avoidance of chemicals reinforce the idea that this condition is not curable and may even contribute to its aggravation and persistence, as systematic avoidance reinforces the aversive conditioning and is against effective desensitization. As to the prayer, if indeed it is effective, it could be related to the hope and the confidence that some solution will come, which will reduce the focus on the issue and bring relief and calm, thus reducing the stress level. This is indeed expected to be effective for a stress-related disorder, such as MCS. There are many reports of spontaneous healing after prayer for various conditions, including medically unexplained symptoms [82].

Some physicians have advocated the use of medications, such as chelators and binders, and other strategies, such as sauna and exercise, to reduce the body burden of chemicals that may have accumulated from chronic low exposure, possibly contributing to MCS [83,84]. As initially mentioned, toxicological studies have not corroborated this hypothesis in MCS patients. Except for some occupational exposures, in most cases, patients have avoided chemicals for a long time because of their long-standing hypervigilance, intolerance, and beliefs. Although sauna may bring general relief, it is not directed at the root causes of MCS.

A placebo effect remains a plausible mechanism for many therapies, which are known to reduce stress brought on by the belief that a recommended therapy is effective. A nocebo effect is the opposite and is related to the belief of a danger associated with something that is not harmful. Van den Bergh et al. have proposed that this mechanism is an important feature of MCS patients [23]. This negative belief may be very important among some patients and may have been reinforced by the repeated experience of physical symptoms triggered by a chemical odor. In our experience, patients who are particularly prone to a nocebo effect are also prone to a placebo effect; they easily believe in beneficial therapies that are not proven, and they easily believe in harmful exposures that are not.

Convincing the patient that his symptoms are not caused by a toxic or allergic mechanism may be sufficient in some cases to stop the reaction, as those reported by Shusterman et al.; in these cases, the explanations were provided shortly after the occurrence of their intolerance, following an accidental toxic exposure. But in most cases, especially in the chronic phase, some form of psychotherapy and other therapies will be needed to reverse the conditioning and the underlying trauma(s) and anxiety trait.

Explaining the mechanisms underlying MCS to the patient remains necessary, although generally not sufficient, but the timing and the manner are crucial. One must avoid confrontation and denigration and use factual and scientific data and obvious contradictions in appropriate moments to raise questions and doubt about erroneous beliefs that impede the resolution of MCS. As mentioned, the immediate physical and uncontrolled nature of the symptoms triggered by a chemical odor is a frequent argument for rejecting the implication of stress and psychological factors. Explaining the pathophysiology of stress (including panic attack, hyperventilation, etc.), function and dysfunction of the autonomous nervous system and the limbic system, trauma memory, and conditioning, using real examples, and using experiences reported by the patient and others, are important to bring an understanding of MCS and comorbidities, and to motivate for the treatment strategies. These explanations may have to be repeated many times during the therapeutic process. Reporting the effectiveness of trauma focus and desensitization psychotherapies for comorbidities may also bring hope for improvement.

In this proposed theoretical framework, a chronologic and thorough understanding of the events and the factors specific to every patient, which may have contributed to the development and aggravation of the MCS, is crucial to identifying the relevant targets for treatment. The type of presentation, according to the three types mentioned, may help to focus on the periods of interest and the main events. In particular, the events occurring at the time of acquisition and aggravation of the reaction to one or various chemicals must be carefully investigated, including in the workplace and personal life. But the memory of relevant events may only come as part of the psychotherapy processes, particularly in the case of amnesia.

The effectiveness of the treatments for PTSD has been well evaluated, and some psychotherapies are clearly considered evidenced-based. As recently mentioned by Ad de Jongh et al., most international clinical practice guidelines recommend EMDR therapy as a first-line treatment for PTSD, with support from more than 30 published randomized controlled trials [85]. Various hypotheses have been proposed to explain its mechanisms. Among these, Ad de Jongh et al. mention that there is support from neurobiological research that taxing the working memory, such as in EMDR, suppresses the activity of the amygdala, which acts as the brain’s “alarm bell” and plays a central role in the storage and reconsolidation of memories. Many EMDR protocols have been developed for other mental disorders and psychosomatic disorders, such as medically unexplained symptoms, but the research is more recent than for PTSD [86]. In a cost-effectiveness analysis of ten treatment modalities for PTSD, EMDR has been found to be the most cost-effective and trauma-focused CBT with the largest evidence base [87]. There is a growing body of evidence or established evidence for other therapies, such as the Emotional Freedom Technique, Somatic experiencing, Coherence therapy, Flash technique, and Internal Family System [88,89,90,91,92]. We must emphasize that no therapy is fully effective for everybody and that the olfactory memory may be particularly persistent. The effectiveness is also related to the time elapsed before starting the treatment of PTSD. Importantly, trauma-focused psychotherapies can contribute to the resolution of the comorbidities sharing the same origins.

In EMDR, after the traumatic events have been successfully addressed and recoded in the memory, the triggers are specifically targeted; therefore, odors of chemicals associated with the traumatic event have to be targeted, as well as the other types of trigger associated with the event(s) [65]. In EMDR, negative thoughts, emotions, and physical reactions associated with the traumatic event are all addressed. In EMDR, in comparison with exposure therapy, the patient does not have to relive the whole traumatic event many times. The Flash technique reduces exposure to traumatic memories even more. Robin Shapiro, an author and clinical psychotherapist, has successfully treated many MCS patients with EMDR 2.0 (which reduces exposure to traumatic memories in comparison to basic EMDR), vagal calming, and visualization exercises [93]. The treatment of MCS may have to be repeated following new stressful or traumatic events or when erroneous beliefs reappear.

The conditions essential for memory reconsolidation and effective psychotherapy have been elaborated by Bruce Ecker et al., creators of Coherence therapy, based on solid scientific grounds [94,95]. These can be briefly summarized as follows: a process by which, following memory reactivation of the trauma, the patient experiences a dissonance between the consolidated information and the new information; in other words, when the new information and experience bring positive opposing views from the previous ones. Research has shown that such a process brings neurophysiological modifications in the brain through the process called memory reconsolidation. As previously mentioned, imaging studies have demonstrated that EMDR and trauma-focused CBT can reverse the abnormalities observed in the structure and function of the brain among PTSD subjects [61]. Not all the psychotherapies and psychotherapists have the potential to fulfill these conditions.

Long-standing hypervigilance related to multiple childhood traumas (such as the case presented previously), complex PTSD, and dissociative disorders can be treated with the same trauma-focused psychotherapies, with adaptations, and usually require prolonged treatments by experienced and specialized therapist(s), although, in a study performed in Amsterdam, the majority of the patients (over 85%) diagnosed with complex PTSD lost their diagnosis after 8 consecutive days of intensive trauma-focused treatment [96]. Other therapies are often used, including body-focused therapies (also called bottom-up approaches rather than top-down) and group therapies. The MCS is more resistant among these patients [97].

Herman Staudenmayer has also used biofeedback to treat MCS with success [97]. Van der Bergh et al. have proposed a detailed stepped therapy protocol including psychoeducation, hyperventilation provocation, exposure to environmental cues, acceptance therapy, home behavioral exercises, and dealing with other stressful conditions [98].

Testimonies from many patients and health professionals are reported on the web in support of the daily practice of self-administered programs, such as the Dynamic Neural Retraining System (DNRS) and the Gupta program [99,100]. As to the DNRS, the results of a one-year follow-up study (unpublished so far) without a control group, carried out in 2016, among 102 subjects with complex chronic illnesses and MCS, has shown an improvement score of 70–90% in five domains, including chemical odor intolerance, which could hardly be explained only by a placebo phenomenon [101]. This program can be useful in association with more formal psychotherapy targeting the underlying traumas and help to maintain good practices to control their negative thoughts and reactions to odors.

Given that emotional traumas can impede the functioning of the autonomous nervous system, the Safe and Sound Protocol (SSP), derived from the polyvagal theory elaborated by Stephen Porges, could be another potentially effective therapy, as it is designed to restore the perception of safety by the autonomous nervous system, which has been altered or lost following traumas [102].

Cognitive approaches alone may not be as effective because these reactions are frequently uncontrolled and unconscious and derive from the autonomous nervous system. This could explain the partial effects of a randomized controlled trial study published by Hauge et al. on the effectiveness of mindfulness-based cognitive therapy [103]. But cognitive approaches are important to motivate the patient for the right treatment and to restructure erroneous beliefs that may interfere with the resolution of MCS along the treatment process.

Anecdotal improvement of MCS has been reported with serotonin-based antidepressants, which can be supported by the mechanism shown in the Hillert et al. study, but no controlled study has been reported [104,105]. Medication, such as anxiolytics and antidepressants, are frequently prescribed for the comorbidities. These medications alone do not resolve the underlying traumas but are often recommended along with psychotherapy. Unfortunately, patients with chronic fatigue syndrome, PTSD, and other stress-related disorders often have an intolerance to various medications because of metabolic disturbances, which cause many side effects and prevent the use of many medications [42].

Herman Staudenmayer has mentioned some factors associated with a poor prognosis [97]. Our clinical experience is somewhat similar, including the following types of patients: those for whom the MCS has evolved over many years or decades, with a severe intolerance and who live in social withdrawal; those who have complex mental disorders associated with important developmental traumas; those who have a low income, which impacts their access to psychotherapy, and who face many stressors; and those who tend to somatization. The prognosis is also poor among those who deny the importance of psychological factors or refuse psychotherapy, who have persistent erroneous beliefs about the toxicity of chemicals, who persistently search for a physical and external cause of their disease, who have an attitude of victimization, or who have attributed a central role in their lives to their illness, and invest much time in advocacy. Some people cannot accept that their beliefs were wrong for so many years. Ultimately, social activities may be impacted to varying degrees, causing difficulties in their relationships and, in the worst cases, withdrawal from society.

Controlled studies should be conducted to confirm and improve the therapies that seem to be effective in a multidisciplinary approach. The types of presentation of MCS could be considered, as well as the prognostic factors mentioned above. For instance, the development type may be considered to have a worse prognosis, as the intolerance may have evolved for decades, with chronic comorbidities and possibly strong negative beliefs acquired. The choice of treatment for the accidental type is more obvious based on the evidenced-based modalities available for PTSD, although one may question the relevance of conducting such a study, given the vast number of studies available. A comorbid condition such as complex PTSD has a poorer prognosis; although the results of the study in Amsterdam have been conclusive, this intensive approach may be difficult to implement elsewhere. Many therapies have been evaluated for comorbid disorders, including panic attacks and phobias, and should be the basis for MCS. Given the neurobiological basis of the study by Hillert et al., the use of serotonin antidepressants should be evaluated more formally, with and without psychotherapy. Given the biological markers and neuroimaging techniques used in research on the etiology of MCS, similar techniques could be used to support the effectiveness of the evaluated therapies.

### 3.5. Prevention

In the occupational setting, the prevention of MCS is related to the prevention of accidental and chronic toxic exposures and the detection of emotional trauma associated with such events. The recognition of toxic or harmful occupational exposures and the avoidance of systematic appeal of occupational claims in such circumstances would greatly reduce the secondary emotional trauma associated with the physical disorder. The quick recognition of an MCS and its referral rather than its denial or contestation would much reduce the chronicity of this disorder.

In the general population, primary prevention of MCS sends back to the prevention of all kinds of violence at all ages, which is a huge task. The public health impact of violence and emotional traumas, particularly in childhood, is often called the silent epidemic because of all the physical and mental associated disorders that may have a huge impact forever [25,26,30]. The medical and psychological services needed to treat all these disorders will never be sufficient.

Intervening as soon as an MCS and a trauma-related disorder are identified, in other words, engaging in tertiary prevention, is also important because the effectiveness of the treatment is partly related to the latency period before treatment. Informing the public about the causes of MCS and the treatments that are recommended versus those that are not may prevent a permanent disability related to this disorder. Advocating only the withdrawal of chemicals without effective treatment is deleterious and conveys the idea that MCS can only be permanent.

## 4. Conclusions

In this analysis, we have shown that MCS can be well characterized, although its manifestations are diverse. A set of eleven diagnostic criteria was derived from the scientific literature and clinical practice.

There is strong documentation supporting its etiology and the underlying mechanisms, with neurobiological abnormalities coherent with the comorbidities associated with MCS. The association with the comorbidities related to anxiety/stress/trauma mechanisms is well demonstrated, and MCS can be viewed as a chronic disorder of the same type. It results from a conscious or unconscious inaccurate interpretation of threat, programmed in the brain, following an innocuous exposure to an odorous substance, which triggers an immediate stress response with various manifestations. It can also trigger or aggravate manifestations related to chronic comorbidities. Traumatic events play an important role in the development of MCS, and various risk factors may contribute to its development. This scientific information constitutes an important guide for selecting the right treatments.

Three common types of presentation that may coexist were identified from the scientific literature on its etiology and the underlying mechanisms, and secondarily from the cases encountered in clinical practice in occupational and environmental medicine. These were summarized as (1) the accidental type, following an initial toxic exposure causing an associated emotional trauma, (2) the associative type, following a repeated innocuous exposure in a threatening context or environment, and (3) the developmental type, following a traumatic childhood/adolescence causing precocious hypervigilance and stress/trauma-related disorders. The mechanisms identified in the scientific literature were applied to real cases representing each type of MCS to explain the factors leading to its development and its resolution. No quantitative analysis was performed to determine these types, but these were determined from the main factors identified in the scientific literature and summarized by Binkley. We suggest that these types may help to structure the variations observed and to point to the circumstances and the events to consider for the treatment. In this sense, their recognition may facilitate the clinical approach and eventually guide the research conducted to evaluate the effectiveness of the treatment strategies.

The understanding of the chronological events and the factors involved in the development of a threatening response to an innocuous exposure to odorous substances, for a specific patient, is an important step for the implementation of a treatment plan. Sharing our understanding of the etiology of MCS and the treatment approach with the patient is a first step, but some patients remain reluctant to the implication of factors other than the chemicals themselves, which is associated with a poor prognosis.

Few studies have been conducted to evaluate specifically the effectiveness of treatments for MCS, but solid grounds exist for the effectiveness of psychotherapies targeting emotional trauma, with support from neurobiological research. The effectiveness of psychotherapies to address some of these comorbidities is also well demonstrated, although improvements are expected. Psychotherapy, which targets the underlying trauma(s), has the potential to be more sustainable and also effective against both the MCS and the stress/trauma-related comorbidities. Research should be conducted to confirm and improve the therapies that seem to be effective, taking into account the types of MCS and its prognosis.

The recognition of this entity is important because MCS may significantly constrain the life of the patient, and it may point to other stress and trauma-related disorders, all having much impact on the health. The term idiopathic should be abandoned, as the etiology and the underlying mechanisms are much better understood.

## Data Availability

The data of the clinical cases described are not publicly available due to privacy and ethical restrictions.
